# Odor Characterization of White Wines Produced from Indigenous Greek Grape Varieties Using the Frequency of Attribute Citation Method with Trained Assessors

**DOI:** 10.3390/foods9101396

**Published:** 2020-10-02

**Authors:** Evangelia Nanou, Emorfili Mavridou, Fotios S. Milienos, Georgios Papadopoulos, Sophie Tempère, Yorgos Kotseridis

**Affiliations:** 1 Laboratory of Oenology & Alcoholic Drinks (LEAD), Department of Food Science & Human Nutrition, Agricultural University of Athens, 75 Iera Odos, 11855 Athens, Greece; enanou@aua.gr (E.N.); emorfili.mvrd@gmail.com (E.M.); 2 Department of Sociology, Panteion University of Social and Political Sciences, 136 Syngrou Avenue, 17671 Athens, Greece; milienos@panteion.gr; 3 Laboratory of Plant Breeding and Biometry, Department of Crop Science, Agricultural University of Athens, 75 Iera Odos, 11855 Athens, Greece; gpapadop@aua.gr; 4 Unité de Recherche Oenologie, EA 4577, USC 1366 INRA, ISVV, Université de Bordeaux, Bordeaux INP, F33882 Villenave d’Ornon, France; sophie.tempere@u-bordeaux.fr

**Keywords:** frequency of attribute citation, CATA, trained panel, correspondence analysis, cluster analysis, sensory analysis, aroma profile, Greek wines

## Abstract

The aim of this study was to investigate the sensory aroma profiles of white wines of the indigenous Greek grape varieties Assyrtiko, Malagousia, Moschofilero, and Roditis. Twenty-three panelists evaluated 17 wines of the aforementioned varieties using the frequency of attribute citation method. Three indices were calculated to assess panel performance in terms of reproducibility. Correspondence analysis and cluster analysis were employed to investigate the sensory space of the wines. Samples of the Roditis variety were characterized mainly by Banana and Vanilla odors; Assyrtiko samples had Earthy, Mushroom, and Nutty odors, as well as Lemon and Honey for some of the samples. Malagousia wines were described as having Lemon, Grapefruit, and Citrus blossom character, and they shared some descriptors with Assyrtiko wines, such as Mushroom and Earthy, and some with Moschofilero samples, i.e., floral and citrus notes. All Moschofilero wines exhibited a floral odor profile: specifically, Rose, Jasmine, or more Citrus blossom-like. Moreover, some Moschofilero samples also revealed a Grapefruit, Lemon, and/or Earthy character, while others expressed Honey notes. In conclusion, despite common characteristics found within varieties, some samples of different varieties exhibited overlapping profiles, and in some cases, samples of the same variety were quite different from each other.

## 1. Introduction

In the last decade, the wine produced from indigenous Greek grape varieties has been increasingly appreciated in the global wine market [1]. Assyrtiko, Malagousia, Moschofilero, and Roditis, as white varieties, and Agiorgitiko and Xinomavro, as red varieties, are gaining ground among other prestigious international grape varieties [2]. Assyrtiko is the most well-known Greek grape variety and initiated the discussion about Greek wine throughout the wine world [3]. Furthermore, it is the first Greek grape variety planted for commercial purposes in other countries, such as Australia, Cyprus, and Lebanon, while new plantings are growing in Italy, South Africa, and the USA. Assyrtiko is believed to have originated from the volcanic island of Santorini, but it has now been planted throughout Greece [3,4]. Assyrtiko wines have been described as having lime, passion fruit, beeswax, flint, and ‘salty’ aromas [5]. Μalagousia is another relatively well-known white variety. This grape variety was saved from extinction: most growers have abandoned it because of its sensitivity to oidium, but its distinctive aroma contributed to its re-plantation. It is planted all over the country and not related to any Protected Designations of Origin (PDOs) [4]. The aromatic profile of its wines ranges from herbal, minty, and citrusy to peachy and tropical, as well as floral [3,6]. Moschofilero is a variety located in northern Peloponnese, and it is the unique variety of the single varietal PDO Mantinia. The aromatic profile of its wines ranges from honeydew, red apple skin, and cantaloupe to grapefruit zest, lemon, and lime [7]. It has also been described as having floral aromas, specifically that of rose petals [6]. Roditis is one of the most planted varieties in Greece, and its aroma has been characterized as citrusy [8], melon [6], and sometimes mineral [6,8].

Wine aroma is modulated by different viticultural parameters, such as the climate and soil of the vineyard; the variety and clone; viticultural practices; winemaking procedures, such as pre-fermentation processing, yeast strain, and alcoholic fermentation conditions; and wine treatments from fining to bottling [9]. However, despite all of these parameters, most wines exhibit a characteristic and distinctive odor according to the grape variety from which they are produced [9]. All of the previously mentioned parameters can enhance or reduce this character.

The aroma profiles of the wines of the aforementioned main Greek commercial grape varieties are described in popular sources, such as books [4,6] and internet blogs [3,7,8]. However, scientific research on the typical characteristics of these varieties is scarce or even lacking. In particular, the wines produced from the aforementioned white grape varieties lack scientific resources that depict their characteristic aromas. In contrast, the literature has focused mainly on the red grape variety Agiorgitiko [10,11,12,13,14]. Nonetheless, there is relatively abundant research on Greek wines in terms of physicochemical analyses that highlight significant characteristics of these wines [15,16,17,18,19].

Conventional descriptive analysis has been used widely in the sensory analysis of wines [20]. However, previous research has pointed out some disadvantages of applying this method to characterize wines. Wines have a very complex aromatic matrix, and it has been proven that people can only recognize a limited number of attributes in a complex matrix [21,22]. Furthermore, Lawless [23] reported that rating intensity is a very difficult task when applied to complex products, such as wines. Thus, in this study, we chose to apply the frequency of attribute citation method. In this method, trained panelists are asked to review a list of attributes and check those that characterize each sample. This method has been applied in the past [24,25] and more recently [26], and it has produced comparable results to those of other descriptive methods, such as sorting tasks, conventional descriptive analysis, and the pivot profile method [24,25,26]. Furthermore, as this method does not require a rating task, cognitive fatigue is reduced; thus, it can be easily applied to wines.

Our research is the first scientific attempt to investigate the aroma profiles of wines of the varieties Assyrtiko, Malagousia, Moschofilero, and Roditis. We focused on the odor characteristics of these varieties and conducted a rigorous scientific study with a trained panel. The aim of this study was to elucidate how these varieties are characterized and differentiated from each other in terms of odor attributes by means of descriptive sensory analysis. We hypothesized that wines of the same variety would share a common sensory space in terms of odor character and that this profile would be distinct for each variety. Finally, we aimed to propose a guide to evaluate the performance of a whole panel and individual panelists in the case of wine typicality evaluation. 

## 2. Materials and Methods 

### 2.1. Wine Samples 

Seventeen commercial white wines from 4 different grape varieties were used for this study, namely, Assyrtiko (3 samples), Malagousia (4 samples), Moschofilero (7 samples), and Roditis (3 samples). Table 1 shows the details of each wine sample, along with their physicochemical characteristics. All of the samples were 2018 vintage, except for ASR4 (2016). Wine samples were chosen according to the suggestions of the National Inter-Professional Organization of Vine and Wine; different places of origin were chosen in order to cover a high degree of variability for each variety. The wines had no contact with wood during their elaboration process. Before the final sensory evaluation by the panel, the wines were controlled by a team of enologists for any flaws in the wine, and all of them were found to be normal. However, some of them were found to be rather ‘tired’ and to have ‘lost’ their character and were thus eliminated before evaluation. Samples were stored in the Laboratory of Oenology and Alcoholic Drinks of the Agricultural University of Athens, and the sensory evaluation took place in December 2019. 

### 2.2. Panelists 

Twenty-three panelists (16 females and 7 males; mean age: 43 years (age range: 21–63 years))—students and staff of the Agricultural University of Athens—participated in the study. They were recruited on the basis of their interest and availability to participate in the study. They were habitual consumers of white wine. All of them passed through a series of screening tests to confirm their ability to take part in wine sensory assessments. Panelists were not paid for their participation.

### 2.3. Training Process 

In total, 21 training sessions of 45 min took place over a period of five months. Panelists were instructed to refrain from eating, drinking, and smoking for 1 h prior to the sessions. Furthermore, they were asked to avoid the use of perfumes or perfumed cosmetics. Panelists attended these sessions over a period of 4 months. Training included the smelling of odor reference standards and describing the odors of wines. 

In particular, during the first 3 sessions, panelists smelled aroma reference standards from an aroma box (Pulltex, Barcelona, Spain) in order to become familiarized with odors that can be found in wine. Furthermore, in the 2nd and 3rd sessions, they described two wines in each session using the wine aroma wheel of Noble et al. [27]. From the 4th training session onwards, a predetermined descriptor list was given to the panelists. This list was compiled after searching the literature for the varieties used in this study. To the best of our knowledge, no previous scientific work investigating the typical aromas of these varieties has been published. Thus, we checked the available grey literature, including blogs [3,5,8], magazines [28], and books [4,6]. 

A common list of 28 descriptors for all varieties was compiled using the aforementioned sources. Furthermore, from the 4th session onwards, different reference standards were presented to the panelists to familiarize them with the vocabulary. In some cases, different recipes for the same attribute were presented in order to narrow the list down to the most appropriate terms for the panel. In sessions 4–11, the first part included reference smelling, and the second part entailed describing two wines. At the end of each session, the results were discussed, and the most cited attributes for each wine were highlighted by the panel leader. In session 12, panelists evaluated 5 wines in booths using Compusense Cloud (Compusense, Guelph, ON, Canada) in order to familiarize them with the evaluation procedure. In sessions 13–18, panelists smelled reference standards during the first part, while, during the second part, they described 2–3 wines that were similar to those used in the study using up to five descriptors. In this stage, they were allowed to modify the list, i.e., to add descriptors that they felt were missing or remove descriptors that they did not find relevant or did not use. Table 2 shows the final list of the 25 descriptors along with odor categories of the attributes that were created by the panel in order to make the list more intuitive. Reference standards are also described in Table 2. References made by natural products were prepared at the beginning of each day in order to maintain their freshness throughout the training session.

In training session 19, panelists were asked to identify blind-coded reference standards. In session 20, a pretest was conducted using 4 wines of the study in duplicate in order to evaluate their performance. Training session 21 included feedback on the previous sessions and the smelling of reference standards. After these last sessions, the panel was deemed successfully trained and continued with the evaluation of the wines.

### 2.4. Wine Evaluation 

The evaluation of the wine samples (17 wines in two replicates, i.e., 34 samples in total) was divided into four sessions of 8 or 9 samples over a period of two weeks. Wine bottles were opened 30 min prior to the test session and were verified to be free of cork taint by the panel leader. Then, 30 mL was poured into transparent INAO wine glasses [29] covered by plastic Petri dishes in order to allow volatiles to move to the headspace. Samples were left to reach room temperature (20 ± 1 °C). The testing room was ventilated and air-conditioned. Samples were evaluated orthonasally in individual booths. Panelists were asked to check 2–5 descriptors from the list that applied to the sample in front of them. A 10 min break was mandatory after the first 5 samples in each session. Between samples, a 1 min break was enforced. Samples were presented with 3-digit blinding codes in a monadic sequence according to a Latin Square Design. Data were collected using Compusense Cloud, Academic Consortium (Compusense, Guelph, ON, Canada).

### 2.5. Data Analysis 

#### 2.5.1. Individual Panelist Performance

The analysis of panel performance was initially focused on assessing panelists’ reproducibility. For carrying out this task, three indices were computed for each of the 23 panelists. Specifically, *n*_1._ and *n*_.1_ denote the numbers of descriptors chosen by the panelist in the first and second replicate, respectively, *n*_11_ denotes the number of common descriptors chosen by the panelist in both replicates, and *n* denotes the total number of tested wines. The indices are defined as (a) *R* = *n*^−1^ Σ(2*n*_11_/(*n*_1._+ *n*_.1_)), wherein the sum is extended over *n* wines; (b) *p*_11_ = *n*^−1^Σ(*n*_11_/*n*_1._); and (c) the average chi-square statistic (under the assumption of independence) from *n* wines. 

The first two indices (i.e., *R* and *p*_11_) have values in the interval [0, 1], and values close to 1 indicate a high reproducibility; these indices mainly focus on the estimation of the probability of choosing a descriptor in the second replicate given that it was chosen at the first replicate. The index denoted by *R* was used previously by Campo et al. [25]. The last index is the mean value of the *n* chi-square distances under the independence assumption between the two replicates; analytically, after computing the values for each panelist and each wine, a contingency table (with two rows and two columns) that included the frequency distribution of the two replicates was generated; then, the chi-square statistic was computed under the assumption of independent evaluations. The index for each panelist is the mean value of *n* resulting chi-square statistics, with large values indicating a lack of independence (reproducibility) between the two replicates. 

In order to identify poor performances among panelists and exclude their evaluations from the dataset, a descriptive analysis was carried out on the three indices, along with a set of statistical tests for detecting outliers (Dixon test, Chi-square test, and Grubbs test, provided by the “outlier” package in r-project [30,31,32,33]). 

#### 2.5.2. Overall Panel Performance

For assessing the overall panel performance, we took the following into account: (a) the effect of the replicate (reproducibility) and product (discrimination ability) on the selection of descriptors, (b) the average reproducibility for each wine, and (c) the distances between the two replications for each wine (reproducibility). A descriptive analysis and methods such as Friedman’s test, Cochran’s Q test [34], and the non-metric multidimensional scaling (Sammon’s non-linear mapping [35,36]) were conducted. 

#### 2.5.3. Product Characterization 

Products’ sensory profiles were first evaluated by descriptive analysis, and for each wine, the most frequently selected descriptors by the panel were identified. Correspondence analysis on a contingency table containing the average citation frequency of descriptors (for the most selected descriptors) was conducted in order to visualize the product and descriptor space. Moreover, the coordinates derived from the correspondence analysis were submitted to cluster analysis. Analytically, the decision about the underlying number of clusters was based on the package “NbClust” [37] using five distance measures (Euclidean, Manhattan, maximum, Canberra, and Minkowski) and six clustering methods (kmeans, ward.D, ward.D2, single, complete, and average) and evaluating any solution in which the number of clusters ranged from 2 to 10. Among the suggested optimal clustering solutions (30 in total), we chose the one suggested by the most combinations (measures and methods). The sensory profile of the derived clusters was further examined by correspondence analysis. All data analyses were run using the R-project [38]. Finally, a spider diagram with the averaged citation proportion of the most cited odor attributes for each cluster was created using Microsoft Excel 2016 (www.microsoft.com, Microsoft, Redmond, WA, USA). In other words, for wines found in the same cluster, the average proportion of citations for each attribute was computed. 

## 3. Results

### 3.1. Individual Panelist Performance

The individual assessment of panel performance included the analysis of the three indices: chi-square, *R*, and *p*_11_ (Table 3, Supplementary Table S1). The minimum values of both *R* and *p*_11_ (0.167) were found for panelist 2, whereas panelist 17 had the minimum value of the chi-square index. It should be mentioned that panelist 21 had the second smallest values of *R* and *p*_11_ (note that these indices are highly correlated) and the third smallest value of the chi-square index; panelist 23 had the best performance according to all three indices. The Dixon test and Grubbs test for outliers (the normality assumption is not rejected at *α* = 5%) support a lack of outliers; this is not the case for the chi-square test (the alternative hypothesis is that the lowest value is an outlier). However, by excluding panelists 2 and 21 from our analysis, all of the tests for *R* and *p*_11_ became non-significant; without these two panelists, the lowest values of *R* and *p*_11_ were 0.215 and 0.242, respectively (both calculated for panelist 16). On that basis of these findings, it was decided that panelists 2 and 21 should be excluded from further analysis. 

### 3.2. Overall Panel Performance

Cochran’s Q test (Table 4) showed that the overall effect of replicate on the descriptors is non-significant (*p* = 0.138), providing us with evidence for the panel’s reproducibility. This is also the case for each descriptor separately, except for Nuts (*p* = 0.004; the descriptor “nuts” was chosen in 11.5% of the cases in the first session and in 18.5% of the cases in the second session). Table 4 also contains the *p*-values for the effect of product on the descriptors; there are significant effects on 16 descriptors (at a 10% significance level), which supports the ability of the panel to discriminate among products. 

The average values of the three indices (chi-square, *R*, and *p*_11_) for each wine can be found in Table 5; the *p*-values from the Friedman’s test show that there is no statistically significant difference among wines (evidence for the panel’s reproducibility). However, it seems that panelists faced some difficulties in reproducing descriptors for MLG4, ASR1, and MSF1, in contrast to MSF9, MSF10, and ASR3, which resulted in large values for the three indices. 

**Figure 1:**
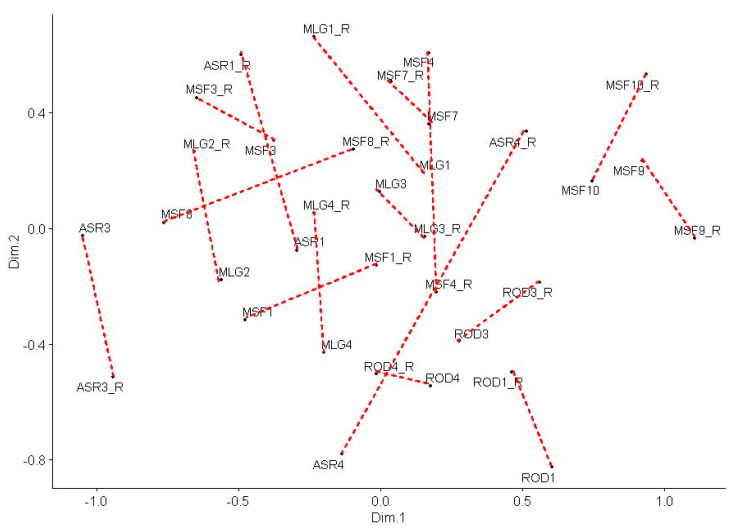
Multidimensional scaling for the first and second evaluation (using the chi-square distance between wines) based on Sammon’s non-linear mapping.

The chi-square distances between the two replications for each wine were assessed by a non-metric multidimensional scaling (based on Sammon’s non-linear mapping). Indeed, the expected small distances between the two replications are confirmed by the results in Figure 1 for most of the 17 wines. The few reproducibility difficulties mentioned above are partially supported by the multidimensional scaling method. 

### 3.3. Product Characterization 

Product sensory profiles were initially evaluated by the frequency of attribute citation. Table 6 shows the six most frequently selected descriptors for each wine. Among the top three most selected descriptors for ROD1, ROD3, and ROD4, we encounter three common descriptors: Banana, Rose, and Citrus blossoms. Lemon, Honey, Nuts, Earthy, and Citrus blossoms are some common descriptors for ASR1, ASR3, and ASR4. Moreover, Rose is the most selected descriptor for MSF3, MSF4, MSF9, and MSF10 (MSF9 and MSF10 have more similarities), whereas MSF1, MSF7, and MSF8 share Lemon and Citrus blossoms among their most frequently attributed descriptors. Some common descriptors for MLG1–MLG4 are Citrus blossoms, Rose, and Lemon. It is worth noting that the most selected descriptors for the wines MSF10 and MSF9, for which we obtained the largest reproducibility indices, were Rose, Citrus blossoms, Honey, and Jasmine. 

**Figure 2:**
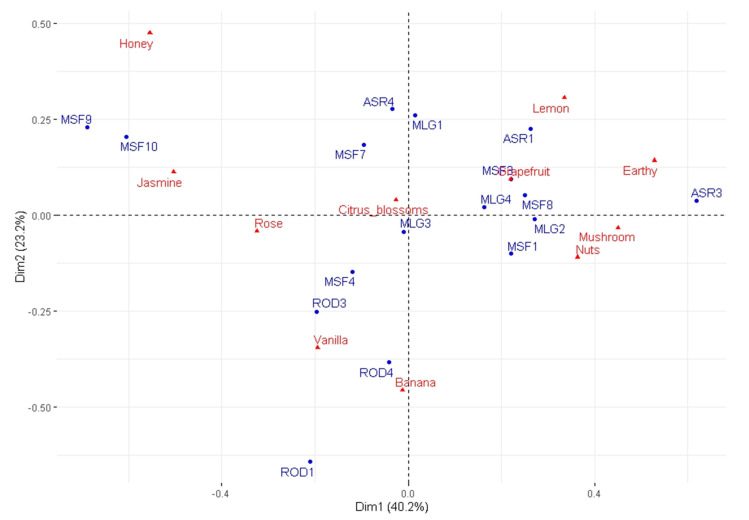
The two dimensions (symmetrical plot) of the correspondence analysis in the product and descriptor space.

A correspondence analysis (the *p*-value from the chi-square test equals 0.04) on a contingency table containing the average citation frequency of descriptors was conducted in order to visualize the product and descriptor space. Specifically, the contingency table was computed based on the descriptors Banana, Citrus blossoms, Earthy, Grapefruit, Honey, Jasmine, Lemon, Mushroom, Nuts, Rose, and Vanilla; these descriptors are among the top three most selected descriptors, as reported in Table 6. Note that although Mushroom is not among the top three most selected descriptors, it was included in the contingency table because it was generally selected many times. Figure 2 shows products and attributes in the first two dimensions of the correspondence analysis (symmetrical plot), which explain 63.4% of the total variability. Therefore, Dim1 is characterized mainly by Lemon, Earthy, Mushroom, and Nuts in the positive part; Honey, Jasmine, and Rose are in the negative part. Dim2 is mainly characterized by Honey and Lemon in the positive part and by Banana and Vanilla in the negative part. 

Among the samples of the variety Roditis, ROD1, ROD3, and ROD4 seem to have a similar profile (also see Table 6), which is mainly characterized by odors such as Banana, Vanilla, and Rose. ASR3 is characterized by Earthy and Nuts, while ASR1 is characterized by Lemon and, to some extent, Grapefruit, which can also be found in the description of ASR3. Among the samples of the Malagousia variety, MLG1 seems to have some distinct elements, such as Honey, while MLG3 is characterized by Citrus blossoms and Grapefruit. Samples of the Moschofilero variety seem to be more dispersed in the sensory space. MSF9 and MSF10 can be described as having Jasmine, Rose, and Honey odors, while MSF8 is described as Lemon and Earthy. 

Moreover, from the residuals of the contingency table (not shown here due to space limitations) and their contribution to the total variability, we can derive the most influential relationships; according to this analysis, these relationships are as follows: (1) Earthy (3.89%) and Nutty (2.71%) odors in ASR3, (2) a Honey (3.40%) odor in ASR4, (3) Rose (2.01%) and Jasmine (4.79%) odors in MSF10, (3) a Honey (5.37%) odor in MSF9, and (4) a Banana (4.60%) odor in ROD1. As can also be seen in Figure 2, ROD1, MSF9, ASR3, and MSF10 are the most influential wines overall. 

**Figure 3:**
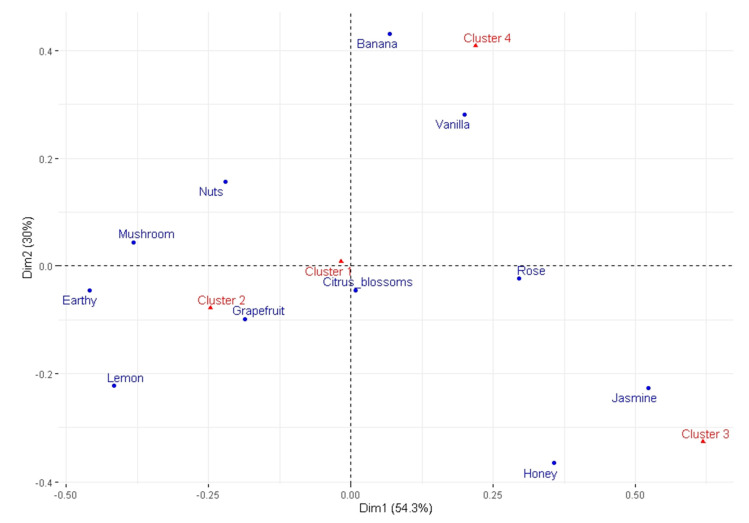
The two dimensions (symmetrical plot) of the correspondence analysis in the cluster and descriptor space.

Cluster analysis, carried out on the first three dimensions (explaining 76.7% of the variability) of the correspondence analysis, indicated the existence of 4 clusters in our set of wines (Table 7). The results show a clear grouping of the three wine samples from the Roditis variety. Additionally, the samples of the Malagousia variety are divided into two groups. Most of the Moschofilero samples are grouped in the first cluster with MLG3. MSF9 and MSF10 are very similar, and both are grouped in cluster 3. Among the Assyrtiko samples, ASR1, ASR3, and ASR4 are grouped in the same cluster. A correspondence analysis on a contingency table, in which rows are determined by descriptors and columns are determined by clusters, was run to visualize the sensory profile of each cluster (also see the Pearson residuals in the last part of Table 7). Hence, Figure 3 shows the products and the cluster space in the first two dimensions (symmetrical plot; explains 84.39% of the variability). A distinction among the four clusters can partially be seen (note the low quality of representation of Cluster 1, which is mainly due to the low representation of citrus blossoms): for example, Cluster 1 is mainly characterized by (in order) Citrus blossoms, Lemon, Banana, and Grapefruit; Cluster 2 is characterized by (in order) Earthy, Lemon, Mushroom, Nuts, and Grapefruit; Cluster 3 is characterized (in order) by Honey, Jasmine, and Rose; and Cluster 4 (in order) is characterized by Banana, Vanilla, Nuts, and Rose. 

**Figure 4:**
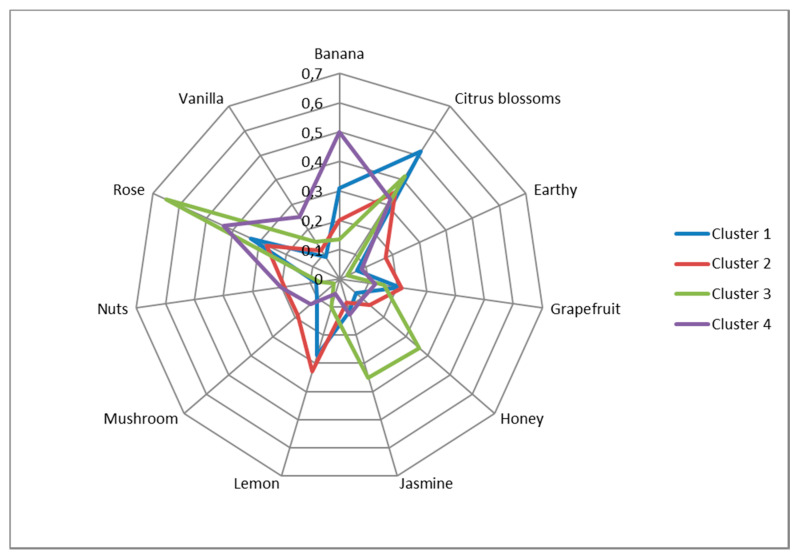
Spider diagram with the average citation proportion of the most cited attributes for each cluster.

Moreover, as shown in Figure 4, the most cited attribute for Cluster 1 is Citrus blossoms (over 50%), followed by Rose (about 33%). In Cluster 2, about 35% of the panelists cited Citrus blossoms; the second most cited term for Cluster 2 is Lemon (about 32%). Cluster 3 shows the maximum proportion of citations for Rose (65%), followed by Citrus blossoms, which was cited by more than 40% of the panelists. Finally, Banana was cited by 50% of the panel in Cluster 4, followed by Rose (over 40%). These results indicate that a flower character prevails in the aroma profiles of Clusters 1 and 3, while Cluster 2 also shows a lemonish character, and Cluster 4 is characterized by a banana aroma. 

## 4. Discussion

In this study, data analysis showed that the panelists had an adequate performance as a whole, after excluding two of them, and individually. The overall performance of the panel was based on the reproducibility of their evaluations and their discrimination ability. The former was evaluated through multidimensional scaling (Figure 1), Cochran’s Q test (Table 4), and Friedman’s test (Table 5), while the latter was assessed through the wine effect on attribute selection by Cochran’s Q test (Table 4). Cochran’s Q test is widely used to analyze binomial data obtained by sensory panels [39,40]. To evaluate individual performance, we proposed the use of three different indices in order to draw more objective conclusions. Thus, we used index R, first used by Campo et al. [25], index p_11_, and the average chi-square statistic for the 17 wines. These analyses showed satisfactory results as well, allowing us to continue with further analysis of our data. 

With regard to the sensory space of products and attributes, Figure 2 reveals a clear character of Banana and Vanilla aromas for the samples of the Roditis variety, as well as Jasmine, Rose, and Honey aromas for the MSF9 and MSF10 samples of the Moschofilero variety. MSF3 and MSF8 are described with a more citrus-fruit-like profile (Lemon, Grapefruit), while the rest of the Moschofilero samples are mainly characterized by Citrus blossoms. The samples of the Assyrtiko variety are associated with aromas such as Honey (ASR4) and Lemon (ASR4, ASR1), as well as Earthy, Mushroom, and Nuts (ASR3). In MLG1–MLG4 samples of the Malagousia variety, Citrus blossoms, Rose, and Lemon are among the top two most cited attributes by the panelists (Table 6). This can also be seen in Figure 2, where, additionally, the odor of Mushroom seems to play a role for MLG2. Interestingly, some samples of different grape varieties, such as ASR4 and MLG1 or ASR1 and MSF3, seem to be more similar to each other rather than to other samples of the variety to which they belong. This is also supported by the results of cluster analysis (Table 7, Figure 3).

Indeed, some clusters are formed by samples of different grape varieties. Specifically, Cluster 1 consists of samples from two varieties, and Cluster 2 comprises samples from three varieties, whereas Clusters 3 and 4 are homogeneous in terms of varietal character. Taking into consideration Figure 3 and Table 7, the odor attributes describing Cluster 1 are Citrus blossoms, Banana, Grapefruit, and Lemon. Cluster 2 is characterized by Grapefruit, Lemon, Nutty, Earthy, and Mushroom odors. Floral aromas such as Jasmine and Rose, as well as Honey, are attributed to Cluster 3. Finally, Banana, Vanilla, and Rose seem to be important attributes for Cluster 4. It should be noted that Citrus blossom seems to be a common characteristic for all clusters—this can also be seen in Table 6, where Citrus blossom is amongst the top three most cited terms for all samples—although, for Cluster 1, it is more important (see Table 7). These characteristics are also evident in the spider diagram (see Figure 4), where Citrus blossom is apparently cited frequently in all of the clusters, but it does not play an important role in the formation of all clusters, especially in Clusters 2 and 4 (see Table 7). Furthermore, although the attributes Earthy, Mushroom, and Nuts are not stressed in the spider diagram, they seem to be important for Cluster 2 in the cluster analysis. 

Regarding cluster formation and varietal origin of the studied wines, Ballester et al. [41], similar to our work, reported that just belonging to a specific category is not enough for an item of this category to be described by the typical characteristics of the category. Furthermore, Rosch and Mervis [42] stated that a typical product can be described not only by the attributes of its own category but also by some attributes of other categories. Within a category, items do have common attributes, but each item does not possess all of the key attributes of that category. Thus, membership in a category has been described as a continuum, where some items may exhibit more typical characteristics than others. Hence, it should not be regarded as an indicator of the absolute inclusion in a category or exclusion from that category [43]. The importance of including samples from different varieties has also been stressed before in order to better explore the limits of the sensory space of a specific variety [41].

Previous research on volatile compounds has revealed the presence of 2-phenylethyl ethanol and phenylethyl acetate in Moschofilero wines [18,44]. These compounds have been further associated with flower aromas, especially rose [45]. This is in congruence with our finding that Moschofilero wines exhibit a floral aroma, including a rose aroma. Moreover, Kechagia et al. [19] found key odorants that are responsible for fruity, honey, and floral aromas in Assyrtiko wines. Accordingly, in our study, Lemon, Grapefruit, and Honey were some of the most cited attributes for Assyrtiko samples. Tyrosol has been previously identified as one of the main phenolic compounds in seven Malagousia wines [46], and it is also known to be responsible for the honey aroma [47]. In our work, we observed that the honey aroma was frequently cited for one Malagousia wine (MLG1), as well as in wines of Assyrtiko (ASR4) and Moschofilero varieties (MSF9, MSF10). Moreover, tropical fruit and banana notes in wines of the Roditis variety have been previously reported [48], but no scientific sources are available on the aroma character of this variety. These correlations from the literature stress the importance of investigating key odor compounds in the study of the characteristic aromas of wines of these grape varieties. 

The present study is, to our knowledge, the first scientific attempt to systematically investigate the sensory aroma profiles of wines of the indigenous Greek grape varieties Assyrtiko, Roditis, Malagousia, and Moschofilero, followed by the assessment of panel performance. Other studies have researched the chemical profiles of some of these varieties and their association with other factors, such as prefermentative treatments [19] and yeast interactions [18]. However, there are no published reports in the literature that aim to identify typical aromas of these varieties. We implemented extensive panel training and a sensory technique, i.e., the frequency of attribute citation, which has been used over the last decade as a reliable and more intuitive alternative to other sensory descriptive methods [24,26,49]. Furthermore, we used three indices to check panel reproducibility, followed by robust statistical analysis, in order to ensure objectivity in our conclusions. Reproducibility indices for binomial data have also been used in prior studies [25,50,51], and they are of indisputable value, as all our data rely on panel assessments. Moreover, we used a common descriptor list for all wines, and no information was given during the evaluation of the variety of each wine so that we could obtain results with no such cognitive bias. However, we should mention that although all samples were of the same vintage, except for one, we could not account for possible different winemaking processes, as these samples were commercial wines from different winemakers and different areas. Another limitation of this study is that the number of samples for the Assyrtiko and Roditis varieties was relatively low to allow firm conclusions regarding their aroma profiles.

## 5. Conclusions

Overall, this study provides a guide to evaluate the performance of a whole panel and individual panelists through statistical indices and analysis. Furthermore, sensory data indicate patterns in the aroma profiles of the wines of four indigenous Greek white grape varieties. Specifically, wine samples of the Roditis variety exhibit mainly a Banana and Vanilla odor profile. Assyrtiko wine samples are characterized by Earthy, Mushroom, and Nutty odors, as well as Lemon and Honey for some of the samples. Malagousia wines are described as having Lemon, Grapefruit, and Citrus blossom characters, and they also share some descriptors with Assyrtiko wines, such as Mushroom and Earthy, and some with Moschofilero samples, i.e., floral and citrus notes. All Moschofilero wine samples exhibit a floral odor profile: specifically, Rose, Jasmine, or more Citrus blossom-like. Moreover, some samples of Moschofilero also reveal a Grapefruit, Lemon, and/or Earthy character, while others express Honey notes. Thus, as is already known, the present work shows that although some descriptors are more characteristic of each of the studied varieties, some samples of different varieties have overlapping profiles, and in some cases, samples of the same variety are quite different from each other.

Future work should focus on using samples that have been vinified with the same protocol. This way, the confounding factor of different winemaking processes will be excluded, and firmer conclusions about aromas that characterize each variety can be drawn. Nonetheless, we should focus not only on finding the key attributes of each variety but also on attributes to define the boundaries of the sensory space of each variety. These findings should ultimately be related and explained by chemical analysis in order to find key odorants that are responsible for those aromas. 

## Figures and Tables

**Table 1:** Wine samples included in the study, along with their physicochemical characteristics *.

Code	Variety	Origin	Vintage	Density (g/mL at 20 °C)	Total Alcoholic Strength by Volume (%vol at 20 °C)	Volatile Acidity (g/L Acetic Acid)	Total Acidity (g/L Tartaric Acid)	pH	Τotal SO_2_ (mg/L)	Residual Sugars (g/L)
ASR1	Assyrtiko	Santorini	2018	0.9894 ± 0.0002	13.7 ± 0.10	0.53 ± 0.04	5.81 ± 0.16	3.07 ± 0.06	73 ± 4	<2
ASR3	Assyrtiko	Macedonia (Drama)	2018	0.9891 ± 0.0002	14 ± 0.20	0.34 ± 0.04	5.59 ± 0.11	3.36 ± 0.07	78 ± 3	<2
ASR4	Assyrtiko	Macedonia (Drama)	2016	0.9882 ± 0.0002	14.4 ± 0.13	0.28 ± 0.03	5.32 ± 0.17	3.28 ± 0.08	71 ± 4	<2
MLG1	Malagousia	Attica	2018	0.9906 ± 0.0003	12.7 ± 0.15	0.28 ± 0.03	5.85 ± 0.11	3.32 ± 0.06	85 ± 3	<2
MLG2	Malagousia	Macedonia (Drama)	2018	0.9893 ± 0.0002	12.1 ± 0.09	0.52± 0.06	5.62 ± 0.15	3.11 ± 0.05	95 ± 4	<2
MLG3	Malagousia	Macedonia (Katerini)	2018	0.9876 ± 0.0002	14 ± 0.18	0.30 ± 0.03	4.95 ± 0.23	3.4 ± 0.05	99 ± 7	<2
MLG4	Malagousia	Evia	2018	0.9905 ± 0.0003	12.9 ± 0.11	0.24 ± 0.02	5.55 ± 0.21	3.49 ± 0.03	79 ± 8	<2
MSF1	Moschofilero	Peloponnese (Arkadia)	2018	0.9901 ± 0.0004	12 ± 0.10	0.15 ± 0.01	6.19 ± 0.25	3.34 ± 0.06	72 ± 5	<2
MSF3	Moschofilero	Peloponnese (Arkadia)	2018	0.9902 ± 0.0003	11.8 ± 0.16	0.11 ± 0.04	6.11 ± 0.16	3.31 ± 0.05	68 ± 4	<2
MSF4	Moschofilero	Peloponnese (Mantineia)	2018	0.9891 ± 0.0002	12.2 ± 0.17	0.12 ± 0.03	5.47 ± 0.18	3.19 ± 0.03	75 ± 6	<2
MSF7	Moschofilero	Peloponnese (Mantineia)	2018	0.9883 ± 0.0002	12.8 ± 0.18	0.23 ± 0.04	5.66 ± 0.17	3.17 ± 0.06	81 ± 5	<2
MSF8	Moschofilero	Peloponnese (Mantineia)	2018	0.9905 ± 0.0002	11.4 ± 0.2	0.17 ± 0.05	6.45 ± 0.18	3.18 ± 0.05	75 ± 4	<2
MSF9	Moschofilero	Peloponnese (Mantineia)	2018	0.9889 ± 0.0002	12.9 ± 0.19	0.34 ± 0.05	4.46 ± 0.13	3.63 ± 0.04	72 ± 6	<2
MSF10	Moschofilero	Peloponnese (Mantineia)	2018	0.9899 ± 0.0002	12.8 ± 0.17	0.4 ± 0.05	5.73 ± 0.16	3.51 ± 0.05	72 ± 4	<2
ROD1	Roditis	Peloponnese (Olympia)	2018	0.9895 ± 0.0002	12.8 ± 0.13	0.22 ± 0.03	4.5 ± 0.21	3.55 ± 0.07	81 ± 5	<2
ROD3	Roditis	Peloponnese (Achaia)	2018	0.9911 ± 0.0004	12.5 ± 0.12	0.17 ± 0.03	5.21 ± 0.15	3.58 ± 0.03	89 ± 7	<2
ROD4	Roditis	Peloponnese (Aigio)	2018	0.9900 ± 0.0003	11.8 ± 0.11	0.29 ± 0.04	5.21 ± 0.14	3.4 ± 0.05	95 ± 4	<2

* All parameters were measured in triplicate. Values are shown as means ± standard deviation.

**Table 2:** Reference standards used during training. The amounts shown were added to 100 mL of white base wine unless otherwise stated.

Odor Category	Descriptor	Reference Standard	Amount
Citrus fruit	Lemon	Lemon peel (fresh)	1 g
	Grapefruit	Grapefruit peel (fresh)	1 g
	Lime	Lime peel (fresh)	0.5 g
	Orange	Orange peel (fresh)	1 g
Tropical fruit	Melon	Standard (Vioryl)	6.25 μL
	Banana	Isoamyl acetate 1g/L (Acros Organics)	3.5 mL
	Pineapple	Pineapple juice (Amita)	25 mL
Other fruit	Apple	Standard (Vioryl)	12 μL
	Pear	Pear (fresh)	Four 2 cm^2^ pieces *
	Peach	Peach juice (Amita)	25 mL
	Apricot	Apricot juice (Amita)	25 mL
Floral	Citrus blossoms	Citrus blossoms water (Alona)	1 mL
	Rose	Rose water (Alikon)	2.5 mL
	Jasmine	Jasmine essence (13% in jojoba oil)	2 drops
Vegetal/Herbaceous	Fresh-cut Grass	cis-3-hexen-1-ol 1g/L (Acros Organics)	4.5 mL
	Mint	Mint (Fino)	1 teabag soaked in 100 mL of hot water for 15 min and then added to the base wine
	Tea	Mountain tea (Evripos)	1 teabag soaked in 100 mL of hot water for 15 min and then added to the base wine
	Chamomile	Chamomile (Evripos)	1 teabag soaked in 100 mL of hot water for 15 min and then added to the base wine
Nuts	Nuts	Hazelnuts (Sklavenitis)	5 g crushed
Earthy	Mushroom	1-octen-3-ol 1g/L (Sigma Aldrich)	3 mL
	Earthy	Wet soil	1 tablespoon *
Caramelized	Honey	Honey (Attiki)	15 mL
	Caramel	Caramel aroma (Vahine)	1.5 g
	Vanilla	Vanille bourbon aroma (Vahine)	1.5 g
Spicy	Pepper	White pepper	3 grains ground *

* Not added to the base wine.

**Table 3:** Descriptive statistics and tests for outliers for the chi-square, *R*, and *p*_11_ indices.

	Index		
	Chi-Square	*R*	*p*_11_
	Descriptive Statistics		
Mean	3.117	0.314	0.327
Std	1.106	0.084	0.087
Min	1.294	0.167	0.167
Max	5.517	0.523	0.527
First quartile	2.419	0.252	0.265
	Correlations		
Chi-Square	1	0.852	0.848
*R*		1	0.979
Normality Test	0.867	0.913	0.802
	Tests for Outliers (*p*-values)		
Chi-square	0.056	0.024	0.039
Dixon	0.968	0.555	0.699
Grubbs	0.584	0.370	0.539

**Table 4:** Assessing replicate and product effects using Cochran’s Q test.

	Replicate Effect			Wine Effect
		Proportions		
	*p*-Value	Replicate 1	Replicate 2	*p*-Value
All attributes	0.138	13.4%	14.2%	0.997
Apple	0.808	12.1%	12.8%	0.873
Apricot	0.617	2.5%	3.3%	0.100
Banana	0.190	25.2%	28.9%	<0.001
Caramel	0.602	5.3%	6.5%	0.068
Chamomile	0.201	2.8%	4.8%	0.005
Citrus blossoms	0.782	38.8%	39.9%	0.096
Earthy	0.233	13.0%	10.4%	<0.001
Fresh-cut Grass	0.241	13.0%	11.3%	0.114
Grapefruit	0.725	17.7%	19.9%	0.273
Honey	0.453	13.0%	15.2%	<0.001
Jasmine	0.691	13.7%	12.2%	0.001
Lemon	0.698	23.6%	24.4%	<0.001
Lime	0.134	11.5%	15.8%	0.007
Melon	0.896	10.9%	10.7%	0.004
Mint	0.705	5.3%	5.7%	0.093
Mushroom	0.112	15.5%	11.3%	0.001
Nuts	0.004	11.5%	18.5%	<0.001
Orange	0.886	9.6%	8.9%	0.478
Peach	0.515	9.9%	11.9%	0.135
Pear	0.876	7.8%	7.7%	0.165
Pepper	0.371	2.8%	3.9%	0.208
Pineapple	0.467	17.4%	16.7%	0.511
Rose	0.253	33.9%	37.5%	<0.001
Tea	0.257	5.9%	3.9%	0.773
Vanilla	0.606	13.0%	14.0%	0.003

**Table 5:** The average values of the three indices (chi-square, *R*, and *p*_11_) for each wine, along with the *p*-values from Friedman’s test.

	Chi-Square	*R*	*p*_11_
ASR1	2.541	0.285	0.309
ASR3	4.031	0.360	0.388
ASR4	3.761	0.321	0.326
MLG1	2.958	0.312	0.334
MLG2	2.492	0.305	0.299
MLG3	3.479	0.314	0.333
MLG4	2.413	0.227	0.239
MSF1	2.533	0.292	0.302
MSF10	6.046	0.472	0.479
MSF3	3.380	0.333	0.366
MSF4	3.535	0.318	0.341
MSF7	3.221	0.350	0.348
MSF8	4.641	0.328	0.354
MSF9	3.315	0.358	0.382
ROD1	3.259	0.343	0.363
ROD3	2.666	0.315	0.329
ROD4	2.960	0.366	0.365
***p*** **-values**	**0.989**	**0.925**	**0.950**

**Table 6:** The six most frequently selected descriptors (proportion of selection in parenthesis) by the panel for each wine.

	Descriptors 1	Descriptors 2	Descriptors 3	Descriptors 4	Descriptors 5	Descriptors 6
ASR1	Lemon (40%)	Citrus_blossoms (33%)	Grapefruit (26%)	Earthy (21%)	Nuts (21%)	Rose (21%)
ASR3	Nuts (36%)	Earthy (33%)	Citrus_blossoms (31%)	Lemon (31%)	Grapefruit (29%)	Mushroom (26%)
ASR4	Honey (38%)	Citrus_blossoms (38%)	Lemon (29%)	Rose (26%)	Mushroom (21%)	Nuts (21%)
MLG1	Rose (33%)	Citrus_blossoms (29%)	Lemon (29%)	Honey (24%)	Earthy (19%)	Peach (19%)
MLG2	Citrus_blossoms (32%)	Rose (29%)	Grapefruit (29%)	Lime (29%)	Fresh-cut_Grass (29%)	Lemon (26%)
MLG3	Citrus_blossoms (48%)	Rose (31%)	Banana (29%)	Grapefruit (29%)	Lemon (29%)	Pineapple (24%)
MLG4	Citrus_blossoms (41%)	Lemon (32%)	Vanilla (26%)	Rose (21%)	Grapefruit (21%)	Mushroom (18%)
MSF1	Citrus_blossoms (57%)	Banana (36%)	Lemon (26%)	Mushroom (24%)	Grapefruit (24%)	Rose (19%)
MSF10	Rose (68%)	Jasmine (41%)	Citrus_blossoms (38%)	Honey (29%)	Grapefruit (21%)	Mint (18%)
MSF3	Rose (36%)	Citrus_blossoms (36%)	Lemon (31%)	Nuts (24%)	Earthy (21%)	Banana (21%)
MSF4	Rose (50%)	Citrus_blossoms (44%)	Banana (35%)	Lime (18%)	Lemon (18%)	Fresh-cut_Grass (15%)
MSF7	Citrus_blossoms (57%)	Lemon (36%)	Rose (33%)	Lime (26%)	Banana (24%)	Apple (19%)
MSF8	Lemon (44%)	Citrus_blossoms (41%)	Rose (35%)	Banana (26%)	Mushroom (24%)	Lime (21%)
MSF9	Rose (62%)	Citrus_blossoms (45%)	Honey (43%)	Jasmine (29%)	Vanilla (24%)	Caramel (19%)
ROD1	Banana (60%)	Rose (45%)	Citrus_blossoms (31%)	Pineapple (31%)	Vanilla (29%)	Melon (19%)
ROD3	Banana (43%)	Rose (38%)	Citrus_blossoms (31%)	Melon (29%)	Pineapple (21%)	Honey (19%)
ROD4	Rose (47%)	Banana (47%)	Citrus_blossoms (35%)	Nuts (32%)	Vanilla (29%)	Mushroom (24%)

**Table 7:** The derived clustering solution and Pearson residuals from the deduced contingency table.

	Cluster 1	Cluster 2	Cluster 3	Cluster 4
	MSF1	ASR1	MSF10	ROD1
	MSF4	ASR3	MSF9	ROD3
	MSF7	ASR4		ROD4
	MLG3	MLG1		
		MLG2		
		MLG4		
		MSF3		
		MSF8		
	Pearson Residuals *			
Citrus blossoms	2.062	−0.850	−0.033	−0.953
Lemon	0.838	2.267	−1.862	−3.007
Banana	0.826	−1.709	−1.828	3.290
Grapefruit	0.672	0.746	−0.800	−1.283
Jasmine	−0.128	−1.594	3.349	−0.092
Rose	−0.263	−1.706	2.578	0.876
Mushroom	−0.703	1.726	−1.914	−0.363
Vanilla	−1.046	−0.766	0.194	2.239
Earthy	−1.326	2.327	−1.733	−0.764
Honey	−1.469	−0.192	3.410	−0.853
Nuts	−1.482	1.306	−1.477	0.821

* The *p*-value for the chi-square test was *p*-value < 0.001.

## Supplementary Materials

The following are available online at https://www.mdpi.com/2304-8158/9/10/1396/s1: Table S1: Chi-Square, *R*, and *p*_11_ values for each of the panelists individually.
