# Supplementary material for: Odor Characterization of White Wines Produced from Indigenous Greek Grape Varieties Using the Frequency of Attribute Citation Method with Trained Assessors
Source: Foods. 2020 Oct 2;9(10):1396. doi: 10.3390/foods9101396 (PMC7600498; doi:10.3390/foods9101396)
Supplement: Supplementary file 1 [file foods-09-01396-s001.pdf]

foods

MDPI

Table S1. Chi-Square, R, and p_{11} values for each of the panelists individually.

Panelist	Chi-Square	*R*	p_{11}
1	4.029	0.429	0.439
2	1.613	0.167	0.167
3	3.149	0.253	0.260
4	3.817	0.338	0.353
5	2.953	0.317	0.314
6	1.860	0.316	0.323
7	4.550	0.430	0.451
8	4.246	0.360	0.333
9	2.723	0.248	0.260
10	2.650	0.291	0.319
11	4.325	0.372	0.409
12	3.146	0.362	0.392
13	3.660	0.344	0.387
14	2.188	0.290	0.283
15	2.810	0.285	0.307
16	1.454	0.215	0.242
17	1.294	0.235	0.248
18	3.664	0.252	0.270
19	3.840	0.347	0.388
20	3.234	0.282	0.314
21	1.526	0.183	0.176
22	3.443	0.380	0.372
23	5.517	0.523	0.527
